# Efficacy and Safety of Bevacizumab for the Treatment of Advanced Hepatocellular Carcinoma: A Systematic Review of Phase II Trials

**DOI:** 10.1371/journal.pone.0049717

**Published:** 2012-12-19

**Authors:** Ping Fang, Jin-hua Hu, Zhi-gang Cheng, Zhe-feng Liu, Jin-liang Wang, Shun-chang Jiao

**Affiliations:** 1 Department of Oncology, Institute of Cancer, PLA General Hospital, Beijing, China; 2 Department of Digestion, Shandong Province Hospital, Jinan, China; 3 Department of Ultrasound Intervention, Institute of Cancer, PLA General Hospital, Beijing, China; University of California Irvine, United States of America

## Abstract

**Background:**

Hepatocellular carcinoma (HCC) is a common cancer associated with a poor prognosis. Bevacizumab is a monoclonal antibody that binds vascular endothelial growth factor, a mediator of tumor angiogenesis. Bevacizumab is currently under investigation as treatment for HCC. We performed a systematic review of the efficacy and safety of bevacizumab for the treatment of advanced HCC.

**Methods:**

PubMed, the Cochrane Library, and Google Scholar were searched using the terms “bevacizumab AND hepatocellular carcinoma AND (advanced OR unresectable)”. Phase II trials of bevacizumab for the treatment of advanced HCC were included. Outcomes of interest included progression-free and overall survival (PFS and OS), tumor response, and toxicities.

**Results:**

A total of 26 records were identified. Of these, 18 were excluded. Hence, eight trials involving 300 patients were included. Bevacizumab was given as monotherapy (n = 1 trial) or in combination with erlotinib (n = 4 trials), capecitabine (n = 1 trial), capecitabine+oxaliplatin (n = 1 trial), or gemcitabine+oxaliplatin (n = 1 trial). Most trials (five of eight) reported median PFS and OS between 5.3 months and 9.0 months and 5.9 and 13.7 months, respectively. The disease control rate was consistent in five of eight trials, ranging from 51.1% to 76.9%. The response and partial response rates ranged from 0 to 23.7%, but were around 20% in four trials. Only one patient had a complete response. Frequently reported Grade 3/4 toxicities were increased aspartate transaminase/alanine transaminase (13%), fatigue (12%), hypertension (10%), diarrhea (8%), and neutropenia (5%). Thirty patients experienced gastrointestinal bleeding (grade 1/2 = 18, grade 3/4 = 12), typically due to esophageal varices.

**Conclusions:**

Bevacizumab shows promise as an effective and tolerable treatment for advanced HCC. The reported efficacy of bevacizumab appears to compare favorably with that of sorafenib, the only currently approved treatment for unresectable HCC. Phase III trials are warranted to comprehensively examine the efficacy and safety of bevacizumab for treatment of advanced HCC.

## Introduction

Hepatocellular carcinoma (HCC) is a very common form of cancer that is typically associated with a poor prognosis. Indeed, worldwide, HCC is the third most common cause of cancer death, as well as being the fifth most frequent cancer in men and the seventh most frequent cancer in women [1], [2]. HCC is particularly common in Asian countries and sub-Saharan Africa, where 80% of worldwide cases occur [1], [3]. However, there is concerning evidence suggesting that the incidence of HCC is increasing in a number of Western countries [1], [4]. Unfortunately, more than 80% of HCC patients present with advanced disease. The treatment options available to these patients are limited and the prognosis is poor [5].

A number of different treatment approaches are available for HCC. Surgical tumor resection is the gold standard of treatment of HCC in patients who do not have advanced disease or extensive comorbidities (including cirrhosis). However, as already noted, most patients are diagnosed with advanced HCC and are therefore not candidates for resection [1], [6]. Liver transplantation is another potentially curative treatment option for HCC, but, similar to surgical resection, is limited to a relatively small proportion of patients [1], [6]. Loco-regional therapies, such as percutaneous ablation and radiofrequency, arterial chemoembolization, and conventional chemotherapies appear to offer limited survival benefits in most cases [1], [6], [7]. Clearly, alternative treatment options are needed.

With our increased understanding of the molecular mechanisms underlying cancer, specific molecular targeted therapies have been developed. One of the key molecular mechanisms underlying HCC is thought to be increased tumor angiogenesis caused by heightened vascular endothelial growth factor (VEGF) signalling [8], [9]. As such, sorafenib, a multikinase inhibitor that blocks VEGF signaling, has been approved for treatment of HCC [1], [6]. This agent has been found to significantly improve time to progression and median survival time compared with placebo in patients with advanced HCC [10], [11]. However, these improvements appear to be relatively modest (approximately three months) [9]. Therefore, other drugs targeting the molecular mechanisms underlying HCC continue to be investigated.

Bevacizumab, a humanized monoclonal antibody that binds VEGF-A [12], been approved for the treatment of various cancers and is currently under investigation as a treatment for HCC [9]. Although the precise mechanism of action is incompletely understood, bevacizumab is thought to decrease tumor vascularity and growth by directly binding with VEGF [13]. This in turn prevents VEGF binding with its receptor and the initiation of the associated angiogenic signal cascade. Bevacizumab may also help normalize tumor vasculature, improving oxygenation and the delivery of cytotoxic drugs [14]. At present, bevacizumab is an accepted treatment for several cancers, notably colorectal, non-small-cell lung, and metastatic breast cancer [13]. A number of Phase II clinical trials have investigated bevacizumab as a treatment for advanced HCC [9]. To date, however, there have been no Phase III trials conducted examining the efficacy and safety of bevacizumab (alone or in combination with other agents) for the treatment of this disease. To gain a better understanding of the efficacy and safety of bevacizumab for the treatment of advanced HCC, we performed a systematic review of the literature reporting findings from relevant Phase II clinical trials.

## Materials and Methods

### Search Strategy

We searched (April 2012) PubMed, the Cochrane Library, and Google Scholar using the terms “bevacizumab AND hepatocellular carcinoma AND (advanced OR unresectable)”. For PubMed, the search was limited to clinical trials, whereas for Google Scholar, the search was limited to identifying words in the title of the article and results that contained at least summaries. We also searched the American Society of Clinical Oncology meeting website for relevant trials.

Abstracts identified in the search were screened for relevancy and duplicate patient databases.

The reference lists of the retrieved articles were hand searched to identify additional relevant articles.

### Selection Criteria

We included articles that described Phase II trials of bevacizumab for the treatment of advanced/unresectable HCC with tumor response outcome measures and toxicities. Meeting abstracts were excluded since they did not report data for the outcomes of interest.

### Data Extraction

Data were extracted by two independent reviewers. Any disagreement between reviewers was resolved by consultation with a third reviewer. Duplicate records were excluded based on review of titles. Abstracts of the remaining articles were reviewed. Studies using duplicate patient data sets and meeting abstracts were excluded. The remaining articles underwent full text review for relevancy and reporting of outcomes of interest.

The following information/data were extracted from the studies where available: bevacizumab treatment details (dose, combination therapies, number of treatment cycles, and line of therapy), Cancer of the Liver Italian Program (CLIP) score, Child-Pugh class, Eastern Cooperative Oncology Group (ECOG) Performance Status, Barcelona Clinic Liver Cancer (BCLC) stage, proportion of patients positive for hepatitis B and C virus (HBV and HCV), complete response (CR) rate, partial response (PR) rate, stable disease (SD) rate, disease control rate (DCR: CR+PR+SD), response rate (RR: CR+PR), progression-free survival (PFS), overall survival (OS), and the incidence of toxicities.

### Outcome Measures

The outcome measures of interest were PFS, OS, tumor response, and toxicities. Tumor response (CR, PR, SD) was evaluated using the Response Evaluation Criteria in Solid Tumors (RECIST) criteria or modified RECIST criteria. Toxicities were categorized using National Cancer Institute criteria. The main outcome measures are summarized using descriptive statistics.

## Results

### Selection of Trials

**Figure 1:**
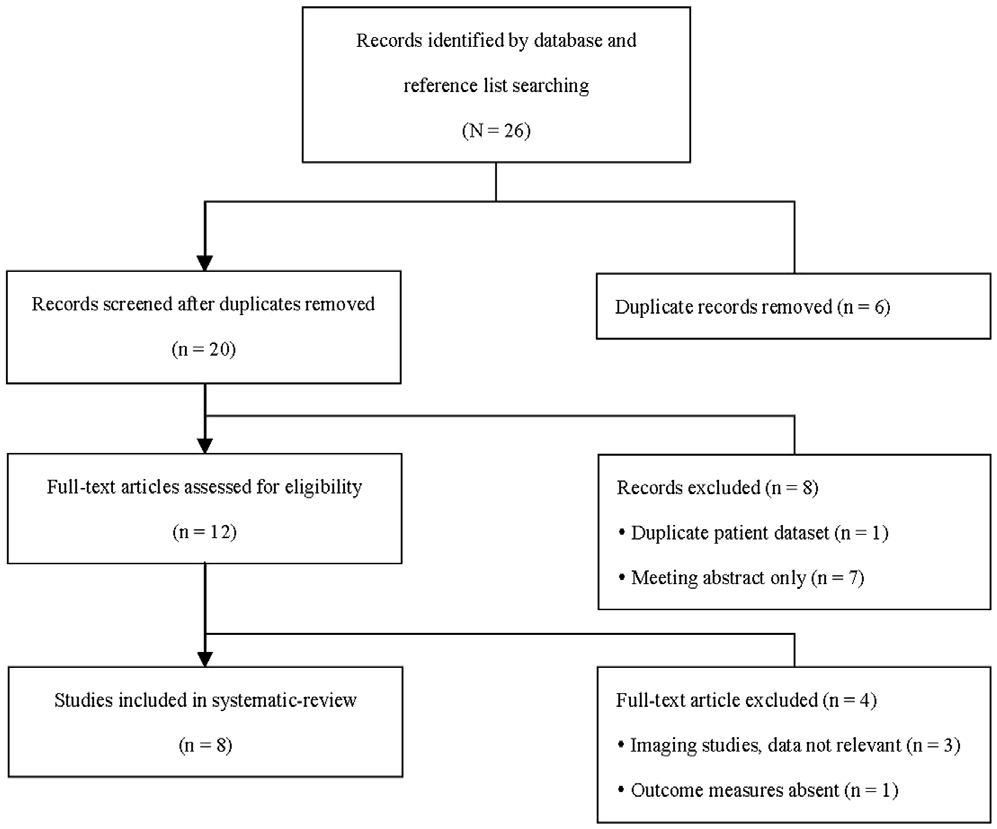
Flow diagram of study selection.

A total of 26 potentially relevant trials were identified in the literature search (Figure 1). After review of the titles, six of these records were found to be duplicates and were excluded. The abstracts of the remaining 20 records were reviewed and a further eight were excluded, including seven meeting abstracts. The remaining 12 records underwent full-text review for assessment of eligibility. A total of eight trials [15]–[22] met the eligibility criteria and were included in the systematic review.

### Patient Characteristics

A total of 300 patients were included in the eight trials (Table 1). The number of patients from individual trials ranged from 10 to 59. All patients had advanced HCC that was unresectable and/or not amenable to loco-regional therapy. For the trials that reported CLIP scores (five of eight trials) [15], [16], [18], [19], 20% to 60% of patients had scores of 3 or 4. For the trials that reported BCLC stages (four of eight trials) [16], [18]–[20], a high proportion (65% to 90%) of patients had stage C cancer. A total of five trials reported on the severity of liver disease using the Child-Pugh classification system [17]–[21]. The majority of patients (58% to 100%) had Child-Pugh class A severity of disease, indicating well-compensated disease. Except for 2 (5%) patients in the trial reported by Sun et al. who had Child-Pugh class C severity [17], the remaining patients had Child-Pugh class B severity of disease. Six of eight trials reported ECOG Performance Status [15], [17], [19]–[22]. The vast majority (95% to 100%) of patients had ECOG scores of 0 or 1, and no patient had a score greater than 2. The proportion of patients with underlying HBV varied considerably, ranging from 7% to 80%. The proportion of patients with underlying HCV was similarly variable, ranging from 0% to 60%.

**Table 1:** Characteristics of trials and patients.

Study	Bevacizumab Dosing	Line	Patients	CLIP	Child- Pugh	ECOG	BCLC	Hepatitis	Cycles of Bevacizumab median (range)
Siegel 2008 [15]	5 mg/kg or 10 mg/kg	1^st^ or 2^nd^ line	46	≤2: 80%	n/a	0/1:95%	n/a	HBV+: 24%	5 mg/kg: 15 (1–48)
	On day 1 of 14-day cycles			3/4: 20%		2: 5%		HCV+: 43%	10 mg/kg: 9 (1–46)
Hsu 2010 [16]	7.5 mg/kg	1^st^ line	45	≤2: 40%	n/a	n/a	B:2%	HBV+: 67%	3 (1–31)
	On day 1 of 21-day cycles			3/4: 60%			C: 98%	HCV+: 18%	
Sun 2011 [17]	5 mg/kg	1^st^ line	40	n/a	A: 57.5%	0/1:95%	n/a	HBV+: 40%	5 (1–38)
	On day 1 of 21-day cycles				B: 37.5%	2: 5.0%		HCV+: 60%	
Kaseb 2012 [18]	10 mg/kg	1^st^ or 2^nd^ line	59	≤2: 51%	A: 86%	n/a	A/B: 24%	HBV+: 17%	86% of patients completed ≥8 cycles
	On day 1 of 14-day cycles			3/4: 49%	B: 14%		C: 76%	HCV+: 29%	
Thomas 2009 [19]	10 mg/kg	1^st^ or 2^nd^ line	40	≤2: 47.5%	A: 87.5%	0/1: 97.5%	A/B:35%	HBV+: 15%	6 (1–13)
	On days 1 & 15 of 28-day cycles			3/4: 52.5%	B: 12.5%	2: 2.5%	C: 65%	HCV+: 25%	
Yau 2012 [20]	10 mg/kg	≥2^nd^ line	10	n/a	A: 100%	0/1: 100%	A/B:10%	HBV+: 80%	3 (2–3)
	On day 1 of 14-day cycles				B: 0%		C: 90%	HCV+: 0%	
Philip 2012 [21]	10 mg/kg	2^nd^ line	27	n/a	A: 74%	0/1: 100%	n/a	HBV+: 7%	2 (1–12)
	On days 1 & 15 of 28-day cycles				B: 26%			HCV+: 53%	
Zhu 2006 [22]	10 mg/kg	≤3^rd^ line	33	median 2, range (0–3)	n/a	median 1, range (0–1)	n/a	HBV+: 18%	3 (1–15)
	On days 1 & 15 of 28-day cycles							HCV+: 30%	

n/a: Not available; CLIP: Cancer of the Liver Italian Program score; ECOG: Eastern Cooperative Oncology Group Performance Status; BCLC: Barcelona Clinic Liver Cancer stage; HBV/HCV: Hepatitis B/C virus.

### Treatment Regimens

Bevacizumab was given as first or second line treatment in all but two [20], [22] trials (Table 1). Bevacizumab was given in combination with erlotinib in four of the eight trials [18]–[21] (Table 2). Bevacizumab was given as a monotherapy in only one trial [15] and was given in combination with capecitabine [16], capecitabine+oxaliplatin [17], or gemcitabine+oxaliplatin [22] in the remaining trials. Bevacizumab was most commonly administered at a dose of 10 mg/kg [15], [18]–[22]. However, a dose of 5 mg/kg was given in two trials [15], [17] and a dose of 7 mg/kg was given in one trial [16]. Dosing typically occurred on day 1 of the dosing cycle (14–21 days) [15]–[18], [20], but occurred on days 1 and 15 in three studies involving 28 day cycles [19], [21], [22]. There was variation in the number of treatment cycles between trials, ranging from a median of 2 cycles to 15 cycles (Table 1). Most trials [16]–[22], however, involved a median of 2 to 6 treatment cycles.

**Table 2:** Summary of efficacy (N = 300 patients).

Study	Treatment	DCR	RR	CR	PR	SD	Median PFS (mo)	Median OS (mo)
Siegel 2008 [15]	Bevacizumab	30 (65.0%)	6 (13.0%)	1 (2.2%)	5 (10.9%)	24 (52.0%)	6.9	12.4
Hsu 2010 [16]	Bevacizumab/capecitabine	23 (51.1%)	4 (8.9%)	0 (0%)	4 (8.9%)	19 (42.2%)	2.7	5.9
Sun 2011 [17]	Bevacizumab/CAPOX	31 (77.5%)	8 (20.0%)	0 (0%)	8 (20.0%)	23 (57.5%)	6.8	9.8
Kaseb 2012 [18]	Bevacizumab/erlotinib	47 (79.6%)	14 (23.7%)	0 (0%)	14 (23.7%)	33 (55.9%)	7.2	13.7
Thomas 2009 [19]	Bevacizumab/erlotinib	27 (67.5%)	10 (25.0%)	0 (0%)	10 (25.0%)	17 (42.5%)	9.0	15.7
Yau 2012 [20]	Bevacizumab/erlotinib	0 (0%)	0 (0%)	0 (0%)	0 (0%)	0 (0%)	1.5	4.4
Philip 2012 [21]	Bevacizumab/erlotinib	12 (44.4%)	1 (2.1%)	0 (0%)	1 (2.1%)	11 (40.7%)	3.0	9.5
Zhu 2006 [22]	Bevacizumab/GEMOX	14 (42.0%)	6 (18.0%)	0 (0%)	6 (18.0%)	8 (24.0%)	5.3	9.6

DCR: Disease control rate (CR+PR+SD); RR: Response rate (CR+PR); CR: Complete response; PR: Partial response; SD: Stable disease; PFS: Progression-free survival; OS: Overall survival; CAPOX: Capecitabine+oxaliplatin; GEMOX: Gemcitabine+oxaliplatin; mo: months.

### Progression-Free and Overall Survival

There was between trial variability in median PFS (Table 2). Median PFS ranged from 1.5 to 9.0 months. The majority of trials (five of eight) reported a median PFS of between 5.3 and 9.0 months (inclusive).

Median OS was less variable than PFS, ranging from 4.4 to 15.7 months (Table 2). The majority of trials (five of eight) reported a median OS of between 5.9 and 13.7 months (inclusive).

### Tumor Response

The DCR was reasonably consistent in five of the eight trials [15]–[19], ranging from 51.1% to 79.6%. The DCR was around 40% in the trials reported by Philip et al. [21] and Zhu et al. [22]. In contrast, Yau et al. [20] reported a DCR of 0%. The RR and PR ranged from 0% to 23.7%, but were close to 20% in four of the eight trials [17]–[19], [22]. Only 1 patient, in the trial reported by Siegel et al. [15], experienced a complete response. Around 50% of patients in six of the eight studies [15]–[19], [21] had SD after treatment. Only 24.0% of patients in the trial reported by Zhu et al. [22] had SD.

### Toxicities

All trials reported on the toxicities experienced during bevacizumab therapy (Table 3). The more common toxicities (experienced by ≥20% of patients, all grades) included nausea and/or vomiting (54%), fatigue (53%), any hemorrhage (46%), diarrhea (44%), increased aspartate transaminase/alanine transaminase (38%), anorexia (37%), dry skin (30%), epistaxis (29%), acne (27%), mucositis (21%), and hypertension (20%). Most toxicities were grade 1 or 2. The most common grade 3 or 4 toxicities (experienced by ≥5% of patients) were increased aspartate transaminase/alanine transaminase (13%), fatigue (12%), hypertension (10%), hemorrhage (8%), diarrhea (8%), and neutropenia (5%).

**Table 3:** Summary of toxicities (N = 8 studies; N = 300 patients).

Toxicity	Studies	Grade 1/2	Grade 3/4	Any Grade
Bleeding				
Epistaxis	4	85 (28%)	1 (<1%)	86 (29%)
GI	4	18 (6%)	12 (4%)	30 (10%)
Thrombosis	1	-	3 (1%)	3 (1%)
Hematochezia	1	1 (<1%)	1 (<1%)	2 (<1%)
Other/Unspecified Hemorrhage	2	10 (3%)	8 (3%)	18 (6%)
Hematological				
Thrombocytopenia	7	42 (14%)	6 (2%)	48 (16%)
Anemia	6	33 (11%)	11 (4%)	44 (15%)
Leukopenia	2	14 (5%)	11 (4%)	25 (8%)
Neutropenia	2	6 (2%)	14 (5%)	20 (7%)
Other laboratory values				
Increased AST/ALT	7	75 (25%)	40 (13%)	115 (38%)
Hyperbilirubinemia	3	35 (12%)	11 (4%)	46 (15%)
Proteinuria	3	40 (13%)	5 (2%)	45 (15%)
Hypomagnesemia	2	35 (12%)	-	35 (12%)
Increased alkaline phosphatase	2	15 (5%)	3 (1%)	18 (6%)
Cardiovascular				
Hypertension	6	28 (9%)	31 (10%)	59 (20%)
Edema	3	20 (7%)	1 (<1%)	21 (7%)
Atrial fibrillation	1	-	1 (<1%)	1 (<1%)
Gastrointestinal				
Nausea and/or vomiting	7	149 (50%)	12 (4%)	161 (54%)
Diarrhea	7	107 (36%)	24 (8%)	131 (44%)
Anorexia	5	104 (35%)	7 (2%)	111 (37%)
Mucositis	5	61 (20%)	2 (<1%)	63 (21%)
Dry mouth	2	56 (19%)	-	56 (19%)
Constipation	4	32 (11%)	1 (<1%)	33 (11%)
Taste alteration	2	32 (11%)	-	32 (11%)
Dysphagia	1	3 (1%)	-	3 (1%)
Gastric perforation	1	-	1 (<1%)	1 (<1%)
Small bowel perforation	1	-	1 (<1%)	1 (<1%)
Dermatological				
Dry skin	2	89 (30%)	-	89 (30%)
Acne	2	76 (25%)	5 (2%)	81 (27%)
Hand-foot syndrome	3	28 (9%)	6 (2%)	34 (11%)
Nail changes	2	31 (10%)	2 (<1%)	33 (11%)
Alopecia	4	32 (11%)	-	32 (11%)
Rash	4	36 (12%)	7 (2%)	43 (14%)
Pruritus	3	22 (7%)	-	22 (7%)
Pain/Neurologic				
Neuropathy	2	43 (14%)	6 (2%)	49 (16%)
Headache	3	42 (14%)	-	42 (14%)
Muscle pain	2	39 (13%)	-	39 (13%)
Abdominal pain	2	27 (9%)	-	27 (9%)
Back ache	1	10 (3%)	-	10 (3%)
Infection				
Wound infection	2	1 (<1%)	3 (1%)	4 (1%)
Lower respiratory tract infection	1	-	1 (<1%)	1 (<1%)
Sepsis	1	-	1 (<1%)	1 (<1%)
Unspecified infection	1	1 (<1%)	1 (<1%)	2 (<1%)
Other				
Fatigue	7	123 (41%)	35 (12%)	158 (53%)
Dry eyes	2	49 (16%)	-	49 (16%)
Weight loss	3	42 (14%)	-	42 (14%)
Ascites	1	3 (1%)	2 (<1%)	5 (2%)
Allergic reaction	1	2 (<1%)	1 (<1%)	3 (1%)
Encephalopathy	1	-	1 (<1%)	1 (<1%)
Fulminant hepatitis	1	-	1 (<1%)	1 (<1%)

GI: Gastrointestinal; AST: Aspartate transaminase; ALT: Alanine transaminase.

Gastrointestinal (GI) bleeding was experienced by a total of 30 patients (grade 1/2 = 18, grade 3/4 = 12) in seven of the eight trials [15]–[20], [22]. Esophageal varices were a commonly reported source of GI bleeding [15]–[19], [22]. Two patients who experienced GI bleeding in the trial reported by Thomas et al. [19] had known portal hypertension.

A total of five patient deaths were reported. In the trial reported by Sun et al. [17], one patient died due to gastric perforation with sepsis. This death was considered secondary to cirrhosis and esophageal varices, but was reported as being probably related treatment. In the trial reported by Siegel et al. [15], one patient died due to treatment-related variceal bleeding. In the trial reported by Zhu et al. [22], one patient died due to respiratory failure. This death was considered possibly related to the gemcitabine and/or oxaliplatin given in combination with bevacizumab. In the trial reported by Thomas et al. [19], one patient died due to complications after experiencing GI bleeding. This patient had known portal hypertension. In the trial reported by Philip et al. [21], one patient died of respiratory failed caused by pneumonia. This death was considered to be unrelated to treatment.

Noted toxicity-related reasons for patient withdrawal included GI bleeding (n = 5) [19], uncontrolled hypertension (n = 3) [15], [22], proteinuria (n = 2) [19], fatigue (n = 1) [19], delayed wound healing (n = 1) [19], and diarrhea (n = 1) [21]. Detailed reasons for patient withdrawal were not available in all studies.

## Discussion

This is the first systematic review to examine the efficacy and safety of bevacizumab for the treatment of advanced HCC. Our review included a total of eight Phase II clinical trials that involved 300 patients. There was a distinct lack of homogeneity among the trials in many respects. However, in general, the results (PFS, OS, tumor response, and toxicities) from these trials indicate that bevacizumab shows promise as an effective and tolerable treatment for advanced HCC.

To date, sorafenib is the only systemic drug therapy approved by the United States Food and Drug administration for the treatment of unresectable HCC. Two Phase III clinical trials have examined the efficacy and safety of sorafenib in patients with advanced HCC. In the SHARP trial [10], median OS in patients treated with sorafenib was 10.7 months, PR was 2%, SD was 71%, and DCR was 43%. In a trial conducted in the Asia-Pacific region [11], median OS in patients treated with sorafenib was 6.5 months, PR was 3.3%, SD was 54.0%, and DCR was 35.3%. Overall, the efficacy of bevacizumab for the treatment of advanced HCC appears to compare favorably with that of sorafenib. Six of the eight bevacizumab Phase II trials reported median OS rates that are similar to or greater than those reported in the SHARP and Asia-Pacific trials [10], [11]. Taken together, these findings are encouraging, and suggest that bevacizumab may prove to be a feasible treatment option for advanced HCC.

Interestingly, none of the patients in the trial reported by Yau et al. [20] responded to treatment. All patients in the trial were refractory to sorafenib and had very advanced HCC. The authors suggested that this lack of a response to sorafenib may have been due to increased proliferation and invasion in the absence of VEGF-related angiogenesis [20]. Given that bevacizumab is also an anti-VEGF therapy, the lack of efficacy is perhaps not completely surprising. This finding suggests that patients who are refractory to sorafenib will receive no benefit from treatment with bevacizumab/erlotinib. It must be noted however, that this trial included a very small number of patients (N = 10). Hence, the results must be interpreted with caution. Further, the findings from Kaseb et al.'s study [18], suggest that patients previously treated with sorafenib (n = 7) may respond to treatment (as indicated by PFS and OS) with bevacizumab given in combination with erlotinib.

There was variability among the studies regarding the dose of bevacizumab given, the co-administered treatment(s), the number of treatment cycles, and the line of treatment. Interestingly, bevacizumab was given in combination with erlotinib, an epidermal growth factor receptor tyrosine (EGFR) kinase inhibitor, in four of the eight studies [18]–[21]. As EGFR is known to promote proliferation of cancer cells [23], dual targeting of VEGF with bevacizumab and EGFR with erlotinib was considered to be a logical treatment approach for advanced HCC. Indeed, the findings from several Phase II studies suggest that erlotinib alone may facilitate disease control [24], [25]. Outcomes were not consistently better or worse with the bevacizumab/erlotinib combination than outcomes associated with the treatment regimens used in other trials. However, the patient populations in the trials involving bevacizumab/erlotinib were far from homogenous, making any meaningful comparison of this treatment combination with other combinations difficult. Clearly, further studies are needed to determine the optimal bevacizumab treatment regimen.

Bevacizumab is known to be associated with a number of toxicities, including nausea/vomiting, diarrhea, fatigue, anorexia, dry skin, hypertension, GI perforation, GI and other bleeding, and thrombosis [26], [27]. There were no unexpected toxicities reported in the Phase II trials of bevacizumab and the vast majority of toxicities were grade 1 or 2 in severity. These findings suggest that bevacizumab was generally well tolerated. Notably, instances of GI bleeding were reported in all but one trial and several patients died due to this complication. The cause of GI bleeding was typically esophageal varices and/or portal hypertension. Patients with these comorbidities (unless controlled in the case of portal hypertension) may not be suitable for treatment with bevacizumab. Indeed, Thomas et al. [19] ultimately implemented a screening process in their trial to identify and exclude patients with esophageal varices before the initiation of treatment. Another notable patient death due to gastric perforation and subsequent sepsis was also associated with esophageal varices (and cirrhosis) [17].

This systematic review has a number of limitations that should be acknowledged. Firstly, none of the trials included were randomized controlled trials. Hence, the evidence from these trials is not of the highest possible quality. Secondly, it is possible that there may be some degree of publication bias in this area of research. We identified several abstracts describing trials that were not further detailed in standard publications; hence, we could not include these trials in the review. Thirdly, there is clearly a multitude of confounding factors (patient and treatment characteristics) that make between trial comparisons difficult. Notably, the severity of concurrent liver disease is likely to have a significant influence on the efficacy of treatment [28]. There were also inconsistencies in intention-to-treat analysis that may have affected outcome data. Finally, few of the studies provided a precise definition of PFS. As previously noted [29], PFS is not infrequently confused with time to progression. Therefore, it is possible that the PFS results extracted from the trials may have actually been a mixture of PFS and time to progression.

In conclusion, the current evidence from Phase II clinical trials suggests that bevacizumab may be a relatively effective and tolerable treatment for advanced HCC. Further, the efficacy of bevacizumab appears to compare favorably with that of sorafenib, the only VEGF inhibitor currently approved for the treatment of HCC. Large scale randomized controlled trials are needed to further investigate this treatment option. In particular, studies are needed to further characterize the efficacy and safety profile, determine which patients are most likely to benefit from treatment, and establish how bevacizumab can be optimally incorporated into HCC treatment strategies.

## Supporting Information

Checklist S1 PRISMA Checklist
(DOC)
